# Spinal muscular atrophy: Should we prescribe approved drugs to cohorts of patients in which they are unproven?

**DOI:** 10.1111/ene.16103

**Published:** 2023-10-25

**Authors:** Lorenzo Fontanelli, Giulia Di Rauso, Gabriele Bellini

**Affiliations:** ^1^ Department of Clinical and Experimental Medicine, Neurology Unit University of Pisa Pisa Italy; ^2^ Department of Biomedical, Metabolic and Neural Sciences University of Modena and Reggio Emilia Modena Italy

## CONFLICT OF INTEREST STATEMENT

Authors have no conflict of interest to declare.

The pivotal study that led to the approval of the first drug for spinal muscular atrophy (SMA) in 2016 was terminated early because of its striking results: more than half of infants receiving nusinersen reached a motor milestone, compared to none of those receiving placebo [1]. Similarly, risdiplam showed impressive results in terms of outcome in toddlers [2]. What both these drugs have in common is that the studies that led to their approval were essentially based on infants with SMA type 1, with evidence, albeit less marked, for children and young adults with SMA types 2 and 3 [3]. Although these studies focused only on a specific portion of the population affected with SMA, the US Food and Drug Administration and the European Medicine Agency approved both treatments without any upper age limit. Nowadays, adult patients are currently treated either with nusinersen or risdiplam, although no randomized clinical trial of efficacy has been conducted in people older than 25 years. As regulatory agencies have not restricted the indications for these drugs, adult patients are fully entitled to receive these potentially beneficial treatments, and to refrain from prescribing them could theoretically result in a denial of cure. On the other hand, the act of prescribing a drug is always the result of a balance of risk and benefits. In this regard, since these drugs are generally well tolerated, and given that the risk is generally low, the risk–benefit ratio favors the benefits even for small and subtle positive effects, and clinicians may decide to opt for the treatment in the hope of a result.

This *bona fide* attitude, however, should be regarded cautiously. Although well tolerated, these drugs are not without side effects [1, 2, 3] and the burden associated with these therapies (i.e., a lumbar puncture every 4 months in the case of nusinersen, or a daily dose of risdiplam) should also be carefully taken into consideration. Of equal importance, these therapies are highly expensive—being in fact two of the most expensive drugs in the world—and their prescription significantly impacts on the financial resources of national health services. The wide range of data obtained by extended prescriptions will undoubtedly shed light on the effect of these molecules in older patients, but this “faculty to prescribe” should not be seen as an “open‐label clinical trial” since such evidence (or, often, “impressions”), which remains outside well‐defined protocols, is less rigorous regarding the outcomes of standardized randomized, double‐blind, clinical trials.

Healthcare professionals should carefully evaluate the available evidence specific to the different patient cohorts in which they are intending to use the therapies; this should include an overall evaluation of the risks and burden of the treatment, and patients should always be informed about current evidence for their specific condition. It is therefore of paramount importance to design randomized clinical trials specifically to assess efficacy outcomes in the older population, as well as to determine international consensus guidance for the use of these innovative treatments in a population with such varied clinical phenotypes.

## AUTHOR CONTRIBUTIONS

**Lorenzo Fontanelli:** Conceptualization; writing – original draft; validation; writing – review and editing; supervision; investigation. **Giulia Di Rauso:** Validation; writing – review and editing. **Gabriele Bellini:** Conceptualization; supervision; writing – original draft; writing – review and editing; investigation.

## Data Availability

Data sharing is not applicable to this article as no new data were created or analyzed in this study.
